# Protracted brain development in a rodent model of extreme longevity

**DOI:** 10.1038/srep11592

**Published:** 2015-06-29

**Authors:** Orsolya K. Penz, Janos Fuzik, Aleksandra B. Kurek, Roman Romanov, John Larson, Thomas J. Park, Tibor Harkany, Erik Keimpema

**Affiliations:** 1 Division of Molecular Neurobiology, Department of Medical Biochemistry & Biophysics, Scheeles väg 1:A1, Karolinska Institutet, SE-17177 Stockholm, Sweden; 2 Department of Molecular Neurosciences, Center for Brain Research, Medical University of Vienna, Spitalgasse 4, A-1090 Vienna, Austria; 3 Department of Biological Sciences, University of Illinois, 840 West Taylor Street, Chicago, IL 60607, USA; 4 Laboratory for Integrative Neuroscience, University of Illinois, 840 West Taylor Street, Chicago, IL 60607, USA; 5 Department of Psychiatry, University of Illinois College of Medicine, 1601 West Taylor Street, Chicago, IL 60612.

## Abstract

Extreme longevity requires the continuous and large-scale adaptation of organ systems to delay senescence. Naked mole rats are the longest-living rodents, whose nervous system likely undergoes life-long adaptive reorganization. Nevertheless, neither the cellular organization of their cerebral cortex nor indices of structural neuronal plasticity along extreme time-scales have been established. We find that adult neurogenesis and neuronal migration are not unusual in naked mole rat brains. Instead, we show the prolonged expression of structural plasticity markers, many recognized as being developmentally controlled, and multi-year-long postnatal neuromorphogenesis and spatial synapse refinement in hippocampal and olfactory structures of the naked mole rat brain. Neurophysiological studies on identified hippocampal neurons demonstrated that morphological differentiation is disconnected from the control of excitability in all neuronal contingents regardless of their ability to self-renew. Overall, we conclude that naked mole rats show an extremely protracted period of brain maturation that may permit plasticity and resilience to neurodegenerative processes over their decades-long life span. This conclusion is consistent with the hypothesis that naked mole rats are neotenous, with retention of juvenile characteristics to permit survival in a hypoxic environment, with extreme longevity a consequence of greatly retarded development.

Within large taxonomic groups, longevity generally correlates with body size and covariates such as basal metabolic rate and brain volume^1^. Exceptions to this rule may indicate species adaptations specific for long life or environmental conditions that, secondarily, can result in extreme longevity. Naked mole rats (*Heterocephalus glaber*), native to Ethiopia, Kenya and Somalia, are the longest-living rodent species known to date. Their maximum life span exceeds 30 years^2^, up to ten times longer than other rodents of comparable body size, even though their weight-specific metabolic rate is one-third less^3^. Recent studies uncovered systemic adaptations in naked mole rats in support of their unusual longevity, including resistance to tumorigenic cell transformation^4^ and forms of age-related neurodegeneration^5^. Despite our increased understanding of the systems physiology of this species, and its clear translational value, data on their brain organization and regenerative potential – encompassing the control of neurogenesis and structural substrates of synaptic neurotransmission – remain largely unexplored.

Naked mole rats live in large colonies in poorly ventilated subterranean burrow systems^2^. Their hypoxic living conditions are associated with a high affinity of hemoglobin to oxygen^6^, low breathing and basal metabolic rates, as well as attenuated hormonal responses^7^ to efficiently utilize limited amounts of oxygen. In addition to these systemic adaptations, we earlier found that isolated brain slices are extremely tolerant to oxygen deprivation *in vitro*^8^. In this regard, naked mole rat brains resemble neonatal rat and mouse brains, leading us to suggest that hypoxia tolerance of naked mole-rats may have evolved by a process of neoteny – a retention of immature characteristics by slowed or arrested development^9^. The neoteny hypothesis for naked mole rats was partially confirmed by developmental studies of NMDA receptor expression^7^ and neuronal calcium uptake during hypoxia^8^ and are consistent with experiments showing high neuregulin-1 levels^10^, stress resistance^11^,^12^, divergent insulin response^13^ and genomic adaptation^13^,^14^ in this species.

Here, we provide evidence that the naked mole rat brain undergoes prolonged postnatal development, including biophysical, molecular, and structural measures of maturation, but not including postnatal neurogenesis^15^,^17^,^18^. First, we studied the size and positioning of adult-born neuronal contingents in olfactory areas and hippocampus throughout the life-span of naked mole rats, cautioning that brain size and neuronal numbers are regulated by sexual activity in this eusocial animal^15^. We found significantly reduced rates of neurogenesis in adult naked mole rats in comparison to adult mouse levels. Despite this reduced neurogenesis, naked mole rat brains continue to grow for at least one year postnatally. We demonstrate the continued and slowed acquisition of mature neuronal morphology, lasting up to at least 10 years postnatally, to build brain volume and neuronal circuits. This is supported by *i*) the presence of axonal pathways, fetal-like guide scaffolds and neurochemical arrangements known to dominate in mouse fetuses^16^, *ii*) continued morphogenesis of hippocampal neurons and *iii*) incomplete synapse segregation in the hippocampus, including mixed subcellular targeting of glutamatergic and GABAergic terminals along the somatodendritic axis of principal neurons. These mechanisms diminish by the second decade of life. In sum, our data identify that developmental processes occurring at the hour-to-day scale in mouse are prolonged along months-to-years time scales in naked mole rats, highlighting that neoteny is a successful evolutionary strategy to enhance the capacity of the central nervous system for successful environmental adaptation to extreme longevity.

## Results

### General remarks: population analysis, age classification and cortical diversification

Naked mole rats are unusual among subterranean mammals in that their colonies are comprised of up to 300 individuals. Within a colony, one female and one to three males are responsible for its reproductive maintenance. Thus, the vast majority of individuals are sexually inactive for their entire lifespan, which can exceed 30 years in captivity^7^,^17^. Therefore, all studies below have been conducted in sexually inactive male naked mole rats, allowing direct comparisons between select postnatal ages, and likely excluding bias associated with hormonal changes. To the best of our knowledge, there is no clear consensus definition for “juvenile”, “adult” or “aged” naked mole rats, whose spontaneous and forced behaviors significantly differ from standard laboratory rodents^18^, and since any such classification in rodents relates to reaching full reproductive capacity. Since the 1-year mark correlates with the onset of sexual activity in reproductive colonies^2^, we assign this age as the inception of adulthood in this species. This is supported by the continued postnatal expansion of brain volume in naked mole rats up to the age of 1 year (Supplementary Fig. 1a,a1). Forebrain-wide mapping data (Supplementary Fig. 1–4) using antibodies that were quality controlled in naked mole rats and compared to mouse tissue equivalents (Supplementary Fig. 1b, 2) are shown as Supplementary Information to support general physiological rules.

### Cell proliferation and apoptosis

**Figure 1:**
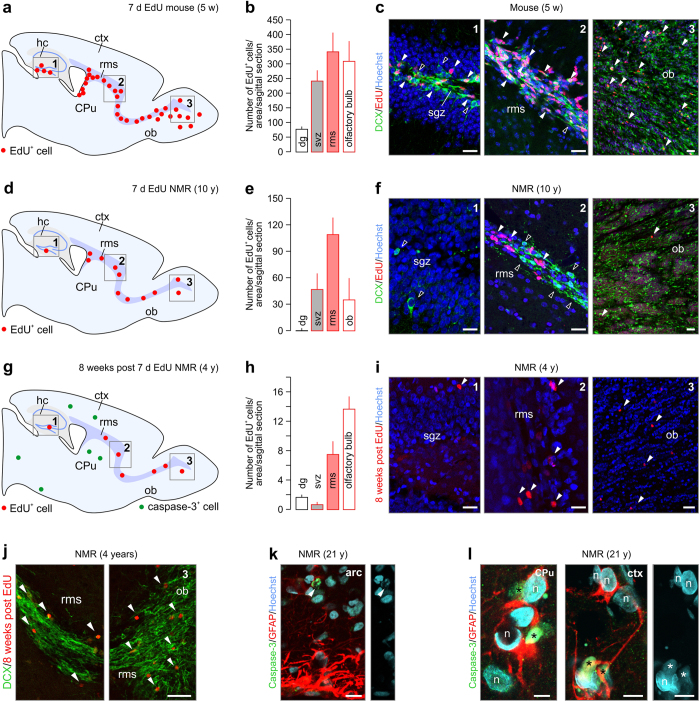
Cell turnover in the naked mole rat. (**a–c**) Analysis of EdU^+^ cells in the hippocampus and svz-rms-ob axis of the adult mouse, pulsed for seven consecutive days with EdU. A sagittal overview showing the two main adult progenitor zones, the hippocampus (grey; 1) and rostral migratory stream (purple; 2,3) in the mouse and naked mole rat brain is depicted in (**a**). EdU^+^ cells are shown in red (*arrowheads*). Doublecortin^+^/EdU^-^ cells (*open arrowheads*) decorated the subgranular zone. (**d–f**) In the 10-year-old naked mole rat, substantially less EdU incorporation is found in the dentate gyrus and svz-rms-ob axis. Note the lack of DCX in the subgranular zone. (**g–i**) EdU^+^ cells were found in increasing quantities in the svz<rms<ob axis 8 weeks post EdU exposure in the naked mole rat. Remarkably, only a few EdU^+^ cells remained as compared to the cohort labeled in (**d–f**). (**j**) Doublecortin^+^ corridor intermingled with EdU^+^ cells in the RMS of the 4-year-old naked mole rat. (**k–l**) Apoptotic cells (cleaved caspase-3^+^; *green circles in (**g**)*) were found scattered throughout the 21-year-old naked mole rat brain, including the arcuate nucleus (*arrowheads*) (**k**), dorsal striatum (CPu) and somatosensory cortex (**l**). Fragmented nuclei (*asterisk*) confirm nuclear condensation. *Abbreviations:* arc, arcuate nucleus; CPu, caudate putamen; ctx, cortex; hc, hippocampus; n, nucleus; ob, olfactory bulb; rms, rostral migratory stream; sgz, subgranular zone; svz, subventricular zone. *Scale bars *= 75 μm (**c,f,I,j**), 20 μm (**k**), 10 μm (**l**).

Neurogenesis is a residual and highly inductive mode for the adult brain to replenish neurons^19^,^20^,^23^,^24^. The rate of neurogenesis is determined by the number of post-mitotic neurons exiting neurogenic niches, their migratory behaviors, and rate of neuronal turnover (that is, the ratio of cell birth and demise). The dentate gyrus^19^,^20^ and subventricular (svz) - rostral migratory stream (rms) - olfactory bulb axis^23^,^24^ are key neurogenic niches in the adult rodent forebrain (Fig. 1a), including not only the mouse^25^ but also the naked mole rat^15^,^26^. Nevertheless, direct determination of cell proliferation in this species, using DNA-incorporating methods, has not been performed yet. We assessed cell proliferation in the hippocampus and the rostral migratory stream, by pulsing 1, 4 and 10-year-old naked mole rats for 7 consecutive days with the proliferation marker 5-ethynyl-2’-deoxyuridine (EdU)^27^,^28^.

In the adult mouse (*n *= 4), EdU-positive(^+^) cells were found in the subgranular zone of the dentate gyrus and the svz-rms-olfactory axis (Fig. 1a–c) and were mostly co-labeled with doublecortin, a microtubule-associated protein expressed in migrating neurons^29^. As compared to adult mouse, a significant decrease in EdU incorporation was observed in the 10-year-old naked mole rat (*n *= 2) svz-rms-olfactory axis (Fig. 1d–f). Strikingly, barely any EdU^+^ signal was detected in the subgranular zone of the dentate gyrus, correlating with minimal amounts of doublecortin immunoreactivity outside of the granular zone (Fig. 1e,f). To investigate if EdU^+^ cells survived in the olfactory bulb, we analyzed EdU incorporation 8 weeks after the last injection (7 consecutive days). Analysis of 1 (*n *= 2) and 4-year-old naked mole rats (*n *= 3) showed an increasing density of EdU^+^ cells towards the olfactory bulb (svz ≪ rms < olfactory bulb; Fig. 1g–i) with only few cells surviving in the olfactory bulb itself (Fig. 1h,j). No difference was seen between 1 and 4-year-old naked mole rats. These data were confirmed by Ki67^30^ immunolabelling, a histochemical marker of proliferation, in the svz-rms-olfactory axis in the 3 months old naked mole rat (Supplementary Fig. 2c). In all adult ages tested (1, 10 and 21-years-old), Ki67 staining was sparsely found in the svz-rms-olfactory axis (Supplementary Fig. 2d) confirming low rates of neurogenesis in postnatal naked mole rats.

Thereafter, we tested, using immunohistochemistry for cleaved caspase-3^31^, if programmed cell death contributes to tissue size control during aging of the naked mole rat. Cleaved caspase-3 was only seen sporadically in adult mice and young (adult) naked mole rats and was particularly absent from the svz-rms-olfactory bulb axis (*data not shown*). Notwithstanding, specimens from a 21-year-old naked mole rat presented a notable density of caspase-3^+^ cells in the arcuate nucleus (Fig. 1k), the lateral caudate putamen and the somatosensory cortex (Fig. 1l). This finding is noteworthy, since cleaved caspase-3 has a short half-life^32^, suggesting prevalent apoptosis in the 21-year-old naked mole rat. In all cases, caspase-3^+^ cells were embedded in a local meshwork of glial fibrillary acidic protein (GFAP)^+^ processes, reflecting focal astrogliosis (Fig. 1k,l). Our data using birth-dating methods show that neurogenesis in naked mole rats, even at reduced rates, is confined to the same neurogenic niches as in common laboratory rodents; and neuronal turnover might be slowed. Therefore, long-lasting structural plasticity could instead be a candidate mechanism to provide neuronal correlates of longevity in this species.

### Neuronal structural plasticity in the dentate gyrus

**Figure 2:**
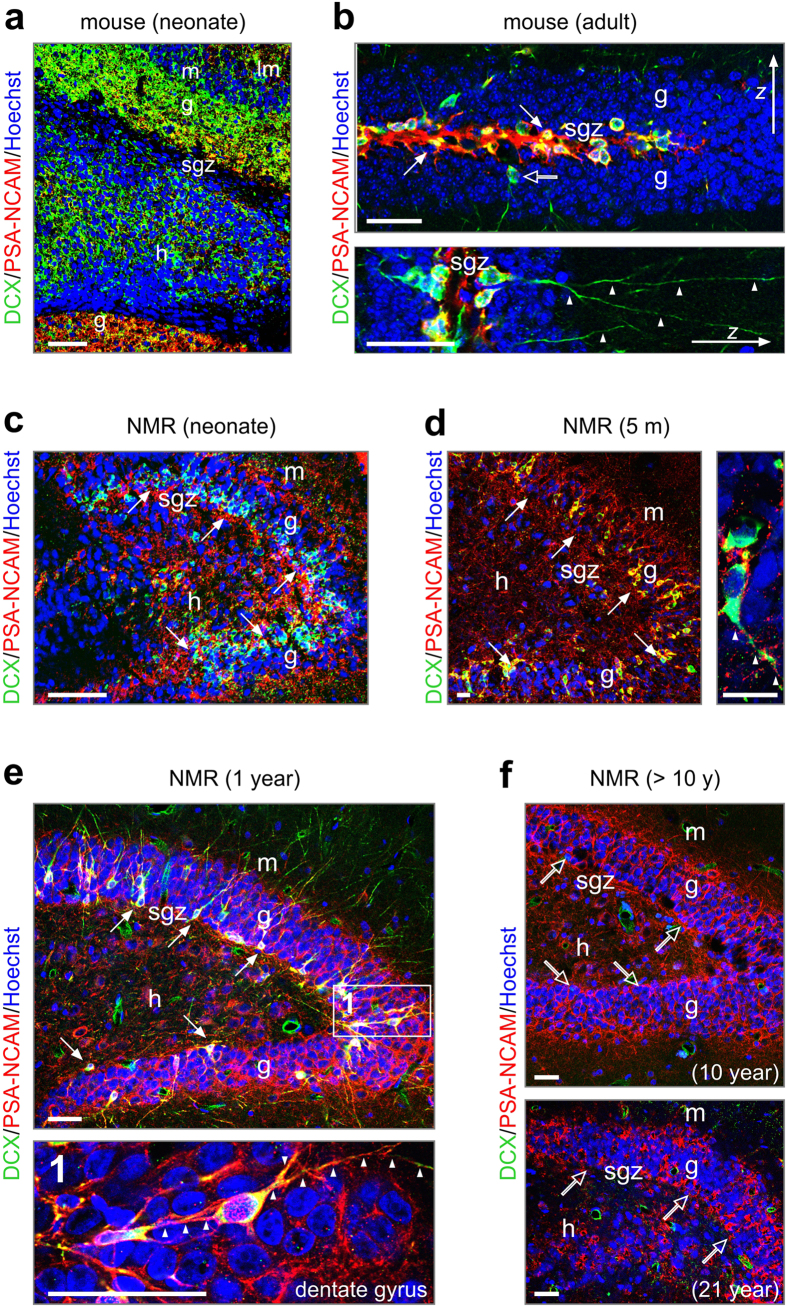
Neurogenesis in the dentate gyrus ceases by 10 years of age in naked mole rat. (**a**) Broadly distributed newborn neurons in the mouse dentate gyrus at birth, as revealed by doublecortin (DCX) immunoreactivity. PSA-NCAM labels motile processes in the dentate blades. (**b**) In the adult mouse, DCX and PSA-NCAM expression are limited to a restricted contingent of new neurons situated in the subgranular zone (*arrows*), with often long dendrite-like processes (*arrowheads*). Open arrows denote DCX^+^/PSA-NCAM^-^ cells exiting the proliferation zone. (**c–e**) In the dentate gyrus of naked mole rats, DCX^+^/PSA-NCAM^+^ cells, were abundant in the subgranular zone until 1 year of age (*arrows*). Many of these cells invaded the granule layer and possessed long, vertical processes (1, *arrowheads*). (**f**) At 10 years of age, DCX expression was largely absent from the subgranular zone (*open arrows*), with residual DCX immunoreactivity restricted to some oval profiles without processes. Likewise, at 21 years of age, DCX^+^ cells were not detected. Note, however, the retained expression of PSA-NCAM, suggesting maintained structural plasticity. Open rectangles denote the general location of insets. *Abbreviations:* g, granule cell layer; h, hilus; lm, lacunosum moleculare layer; m, molecular layer; sgz, subgranular zone; *Scale bars *= 100 μm (**a,c**), 50 μm (**b,e,f**), 20 μm (**d**).

Adult-born neurons recapitulate the cellular developmental program, including the expression of proteins such as doublecortin^29^, as well as negatively regulating cell-cell interactions to allow for sustained structural plasticity in an adult environment (e.g., polysyalilated-neural cell adhesion molecule; PSA-NCAM^33^,^34^). For naked mole rats, their harsh living-environment, as well as their asexuality, might provoke an arrangement in which fetal-like neuronal structural plasticity is retained long after birth to maintain latent “readiness” of neurons for input-specific refinement and wiring (that is, a slowed increase in dendritic complexity and segregation of afferent synapses). Here, we histochemically tested the hypothesis that dentate granule cells (GCs)^35^ (Fig. 2) retain juvenile molecular marks even at advanced ages in the svz-rms-olfactory axis (Supplementary Fig. 3,4).

In neonatal mice (Fig. 2a), doublecortin was abundantly expressed across the cell-dense granule layer, as well as by hilar neurons. Likewise, PSA-NCAM immunoreactivity decorated both blades of their dentate gyrus (Fig. 2a). In adult mice, both doublecortin and PSA-NCAM expression were restricted to the subgranular zone. Doublecortin primarily localized to the somatodendritic axis of adult-born GCs exiting the proliferative zone (Fig. 2b). In contrast, PSA-NCAM appeared more profuse, with appreciable immunoreactivity in the subgranular zone itself (Fig. 2b).

In neonatal naked mole rats, doublecortin immunoreactivity was confined to a band in the innermost third of the granule cell layer, while PSA-NCAM^+^ processes were seen throughout the hilus, granule cell and molecular layers (Fig. 2c). Unlike in mouse, doublecortin^+^ perikarya were not only seen in the subgranular zone but also in the inner half of both blades of the dentate gyrus in naked mole rats aged 3–5 months (Fig. 2d). We attribute these cells to a migrating newborn neuronal cohort given their bipolar morphology (Fig. 2d), apparently small soma size, and their axial process navigating amongst stationary GCs. Unexpectedly, not only these cells but also many doublecortin^-^ processes and perikarya were PSA-NCAM^+^ in the granule cell layer. Similarly widespread doublecortin expression was seen in 1- (Fig. 2e) and 4-year-old (*data not shown*) naked mole rats. The widespread distribution of PSA-NCAM^+^ fibrous structures, likely dendrites and/or axons, at this developmental window suggests permissive environments for cell motility^36^ throughout the dentate gyrus. In contrast, we detected limited amounts of doublecortin immunoreactivity in 10 year or older (Figs 1d–f,2f) naked mole rats.

The above, together with our EdU pulsing data, suggests that the lower rate of postnatal neurogenesis and neuronal migration compared to mice decreased substantially by mid-life in this species. Yet residual PSA-NCAM immunoreactivity implies that the naked mole rat brain retains substantial structural plasticity of immature neurons even if neurogenesis no longer occurs. Notably, similar considerations apply to the svz-rms-olfactory bulb axis, where a pronounced decrease of doublecortin^+^ and/or secretagogin^+^ 37,38,39 migratory neuroblasts was documented by 10 years of age (Supplementary Fig. 4a-h). These observations suggest common temporal rules for postnatal neuronal migration in the naked mole rat forebrain.

### Retention of cortical guide scaffolds

**Figure 3:**
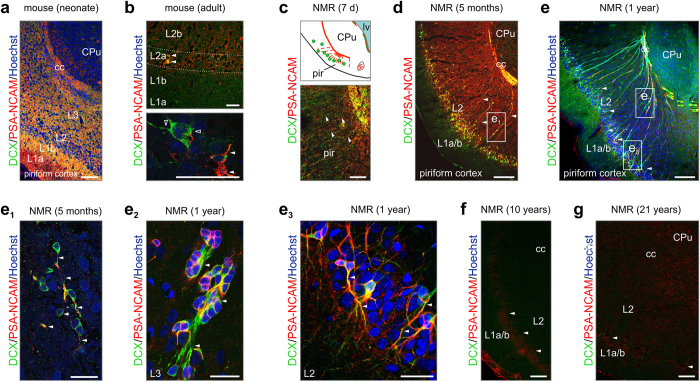
Structural plasticity in the piriform cortex of naked mole rats. (**a**) PSA-NCAM and DCX are widely expressed in the piriform cortex of neonatal mice. (**b**) In the adult mouse, DCX^+^/PSA-NCAM^+^ (*arrowheads*), as well as DCX^+^/PSA-NCAM^-^ (*open arrowheads)*, are found scattered throughout layer 2a of the piriform cortex. Note, however, that DCX^+^ cells in the adult brain can be mature instead of progenitors^16^. (**c,d**) In the young naked mole rat, a large continuum of DCX^+^ cells (e_1_, *arrowheads*) was found embedded in a PSA-NCAM-containing region. (**e–e**_**3**_) At 1 year of age, PSA-NCAM immunoreactivity was diminished (**e**) while DCX^+^ neurons possessed long projections (e_2_,e_3_). (**f,g**) In the piriform cortex of 10 (**f**) and 21 year old (**g**) naked mole rats, PSA-NCAM was strongly reduced (*arrowheads*), while DCX was absent. *Abbreviations:* cc, corpus callosum; CPu, caudate putamen; g, granule cell layer; h, hilus; L, layer; lv, lateral ventricle; pir, piriform cortex. *Scale bars *= 100 μm (**a,d,e,f,g**), 40 μm (**b,c**), 20 μm (**e**_**1**_**,e**_**2**_**,e**_**3**_).

The low rate of neurogenesis in the postnatal naked mole rat brain, particularly the olfactory system, suggests that cellular arrangements to support sensory adaptation over the extended life span of this species might have evolved to offset olfactory neurogenesis. The piriform cortex, where olfactory (mitral cell) axons terminate, harbors doublecortin^+^ neurons in postnatal rodents^16^,^40^ and primates, suggesting extended structural plasticity in this paleocortical multimodal relay station. By relying on our comparative approach, we first show the abundance of doublecortin^+^ and/or PSA-NCAM^+^ neuronal substrates, particularly processes stratified across all layers of the piriform cortex in neonatal mice (Fig. 3a). This arrangement seems temporally restricted since either marker is only and sparsely detected in layer 2a of adult mice (3 months of age; Fig. 3b).

In contrast, we find a scaffold of doublecortin^+^/PSA-NCAM^+^ processes between the white matter (Fig. 3c) and the apical surface of the brain, and spaced equally across the piriform cortex of new born (Fig. 3c), juvenile (5 m, Fig. 3d) and 1-year-old (Fig. 3e) naked mole rats. These processes, likely axons, progressively fasciculate over time and rarely, if at all, contact the brain surface, making a radial glia origin unlikely. High-resolution microscopy revealed that doublecortin^+^/PSA-NCAM^+^ neurons with a bipolar morphology (that is, oval-shaped somata and surface-oriented leading process; Fig. 3e_1_) were in close contact with these “guide structures” in juvenile subjects. Similarly, doublecortin^+^/PSA-NCAM^+^ neurons adopting a pearl-lace-like configuration remained associated to this scaffold in layers 2b/3 (L2b/3) (Fig. 3e_2_). The doublecortin^+^/PSA-NCAM^+^ neuronal contingent in upper L2a was morphologically more heterogeneous with many cells exhibiting ramifying smooth processes (Fig. 3e_3_). These neurons were interspersed in the cell-dense L2a of the piriform cortex, suggesting a form of neonatal murine-like structural plasticity (see also Fig. 3a) that might be retained in naked mole rats over extended postnatal periods.

Conspicuously, we showed the lack of doublecortin immunoreactivity by and beyond 10 years of age with only residual PSA-NCAM immunosignal detected at the L1/2 boundary (Fig. 3f,g). These data, supported by the loss of these structural plasticity markers in the olfactory bulb by 10 years of age (Supplementary Fig. 4j,h) suggest prolonged cellular reorganization as a means to retain neuroadaptation in the olfactory system, including primary cortical areas, of naked mole rats.

### Protracted neuronal morphogenesis in the postnatal hippocampus

Considering the above results and their subterranean lifestyle, one can hypothesize that incomplete neuronal morphogenesis would allow minimal essential synaptic neurotransmission, energy expenditure and exploit hypoxia tolerance found in juvenile mammals. Nevertheless, the extent and time-course of postnatal neuronal morphogenesis in naked mole rats require elucidation. We used correlated neurophysiology and morphometric analysis that focused on CA1 pyramidal neurons and GCs of the hippocampal formation whose molecular and functional homogeneity^41^,^42^ allows the collection of statistically validated data from small cohorts of live animals.

**Figure 4:**
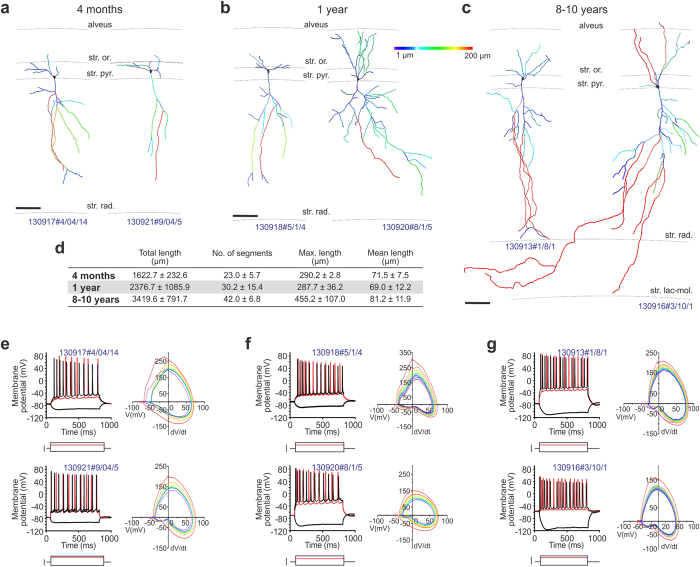
Dendritic morphology and biophysical properties of CA1 pyramidal cells in naked mole rats. (**a–c)** Three-dimensional quantitative morphometry (Imaris) of biocytin-filled neurons in the CA1 pyramidal layer of naked mole rats aged 4 months (**a,e**), 1 year (**b,f**) or >8 years (**c,g**). Black lines denote the hippocampal laminae. Pseudo-colors correspond to the length of dendrite segments (colorimetric scale from 1 μm–200 μm). *Scale bars* = 50 μm. (**d**) Morphometric parameters expressed as means ± S.D. for CA1 pyramidal cells at the age groups indicted. (**e–g**) Current clamp recordings with the most hyperpolarizing and depolarizing current steps depicted in black. Membrane potential traces shown in red were evoked by a depolarizing current step at evoked 2x threshold (in red). *Scale bars *= 150 pA. Phase diagrams of action potentials evoked at 2x threshold. The first action potential was coded red with subsequent traces progressively shaded from warm to cool colors, ending in blue. Note that the threshold potential variations (colored lines) tighten in aging animals, suggesting diminished variations.

Earlier studies showed that the characteristic dendrite architecture of pyramidal neurons develops either *in utero* or early postnatally in both mice and primates^42^,^43^. Here, we studied the somatodendritic morphology of pyramidal neurons (Fig. 4a–c) by reconstructing dendritic arbors in biocytin-filled CA1 neurons. In 4-month-old animals (Fig. 4a), pyramidal-like neurons exhibited rudimentary apical and basal dendritic profiles in the strata oriens and radiatum, respectively; characterized by a short apical dendrite stem and few secondary dendrites that only infrequently ramified. By 1 year of age (Fig. 4b), the dendritic arbor significantly expanded in length and complexity, including the appearance of tertiary dendrites. Apical dendrites in 4-year-old naked mole rats spanned the entire depth of the stratum radiatum, and intruded occasionally into the stratum lacunosum moleculare (Fig. 4c). Quantitative analysis (Fig. 4d) confirmed the above morphological changes, which included not only the gradual elongation of individual dendrite segments but also an increment in the maximal and total dendrite lengths, and the number of dendrite segments.

**Figure 5:**
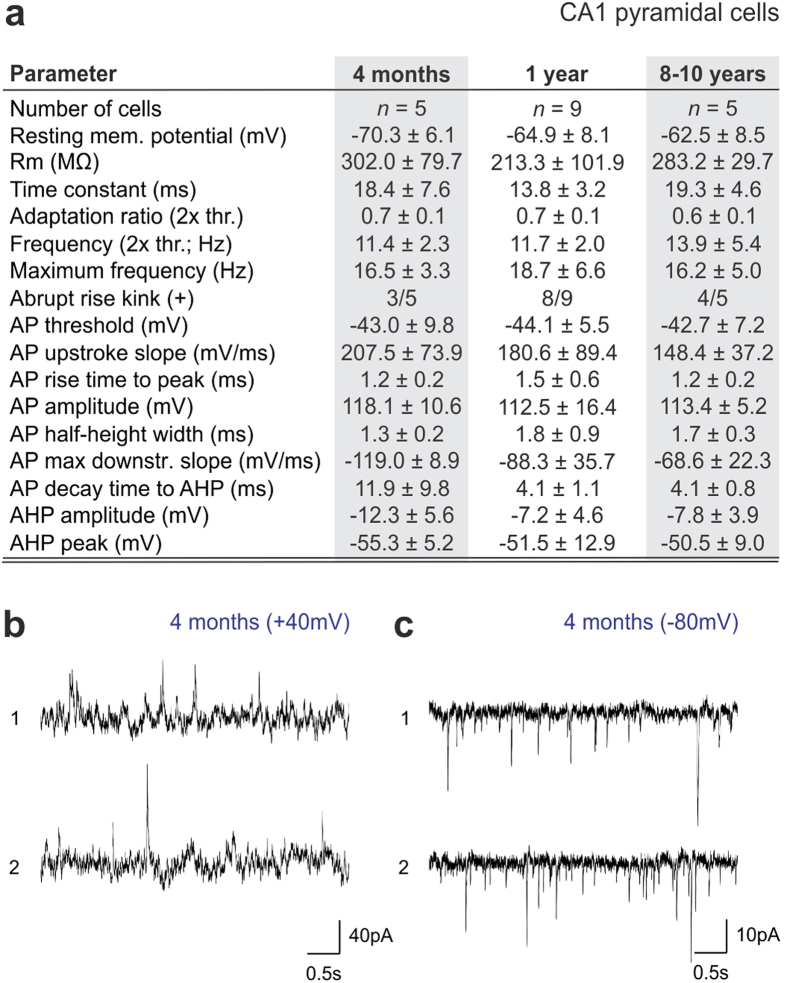
Biophysical parameters of CA1 pyramidal cells in naked mole rats. (**a**) Neurophysiological characteristics of CA1 pyramidal cells (*n *= 5–9 cells from a total of 3 animals/age). (**b,c**) Representative spontaneous activity traces obtained at +40 mV and −80 mV in 4-month-old naked mole rats reveals inhibitory and excitatory inputs, respectively.

Next, we correlated the intrinsic electrical activity of CA1 pyramidal cells with their morphology by analyzing records from whole-cell patch-clamp experiments. We used an intracellular recording solution (see *Materials and Methods*) that produces normal action potential (AP) firing (+40–45 mV of AP peak; Supplementary Fig. 5a) in juvenile (postnatal day 19–23) C57Bl/6 mice. In naked mole rats, our recordings revealed unexpectedly large (up to +80 mV) APs ranging 11–16 Hz in frequency at all age groups studied (*n *= 5 [4 months], *n *= 9 [1 year], *n *= 5 cells [8–10 years]; Fig. 4e-g, Fig. 5a). An apparent decrease in time delay to the first AP together with an increased reliability to fire AP trains at constant frequency was seen in older naked mole rats. Next, we analyzed the variation of subsequent APs at 2x threshold stimulation^44^, to test the contributions of initial segment *vs*. somatic spike inductions. We find that the number of CA1 pyramidal cells with abrupt AP rise (kink)^45^,^46^ increases with advancing age (Fig. 5a), which coincides with a progressive tightening of the AP threshold variation as shown in phase plots in Fig. 4e–g. As such, the input resistance, up-stroke and down-stroke slopes of APs, their decay time to afterhyperpolarization (AHP) and the amplitude of AHP differed considerably between young (4 months of age) and older (1 or 8–10 years of age) CA1 neurons in naked mole rats (Fig. 5a). We used voltage-clamp recordings to assess whether CA1 pyramidal cells in 4-month-old naked mole rats, which morphologically resembled immature neurons (Fig. 4a), received synaptic inputs. By changing the holding potential above or below the calculated reversal potential for GABA (Fig. 4b) and glutamate (Fig. 4c), that is to only record EPSCs and IPSCs graphically, we find mixed inputs to CA1 pyramidal cells during early postnatal development.

We interpret the above data as the continued postnatal maturation of pyramidal cells in naked mole rats, since our recordings show an increased likelihood of AP generation and the stability of AP waveforms in older animals, which are consistent with previous reports on the neurophysiological maturation of pyramidal cells in juvenile mammals along shorter time-scales^43^,^47^. Yet APs were similar throughout all ages suggesting that these cells are functional and able to contribute to the circuitry, with their morphological immaturity perhaps limiting the complexity of circuit power. Since the molecular underpinnings, particularly cation channel composition, in naked mole rat pyramidal neurons remains elusive, we can only hypothesize that the excessive AP overshoot seen here might lie in structural and functional differences of Na_v_ and K_v_ channels^48^.

**Figure 6:**
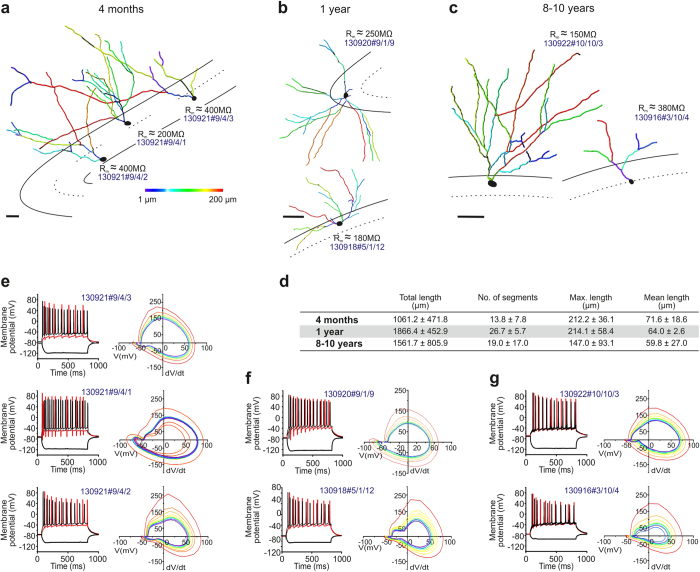
Dendritic morphology and biophysical properties of dentate granule cells in naked mole rats. (**a,b,c**) Three-dimensional quantitative morphometry (Imaris) of biocytin-filled neurons in the dentate gyrus of naked mole rats aged 4 months (**a,e**), 1 year (**b,f**) or >8 years (**c,g**). Dashed black lines denote the boundaries of the granule and molecular layers in the dentate gyrus (upper saber). Pseudo-colors correspond to the length of dendrite segments (colorimetric scale from 1 μm −200 μm). *Scale bars *= 50 μm (**a**), 25 μm (**b,c**). Note that higher resistances (R_m_) correlate with cells deeper in the granule layer and indicate immature neurons. (**d**) Morphometric parameters expressed as means ± S.D. for dentate granule cells at the age groups indicted. (**e–g**) Current clamp recordings of the reconstructed granule cells with the most hyperpolarizing and depolarizing current steps depicted in black. Membrane potential traces shown in red evoked by a depolarizing current step at 2x threshold (in red). *Scale bars *= 150 pA. Phase diagrams of the action potentials evoked at 2x threshold. The first action potential phase diagram was colored red with subsequent traces progressively shaded from warm to cool colors, ending in blue.

Then, we sought to determine whether similar morphological and neurophysiological indices of postnatal development occur in dentate GCs, particularly since we and others^15^,^26^ find low rates of neurogenesis in this region. As such, the dendritic complexity, membrane resistance and resting membrane potential of dentate GCs was dependent on their somatic position in the granule cell layer (Fig. 6a). This is compatible with data in mouse showing an inside-out maturation pattern of GCs, with the ones most distant from the subgranular zone being the oldest neurons^20^,^35^,^49^,^50^. These positional/presumed age differences likely were a factor in the variability of our population records (Fig. 6a–d). As such, we see an almost doubling of the extent of the dendritic arbor and the number of dendrite segments, conferring increased complexity, in 1- and 10-year-old naked mole rats relative to more juvenile ones (Fig. 6d). Yet even in 10-year-old animals we observe rudimentary dendritic arbors of deep-layer GCs (Fig. 6d) that reconcile our doublecortin data and suggest that the slowed migration of these GCs could limit the age-dependent complexity of neuronal networks in this hippocampal area.

**Figure 7:**
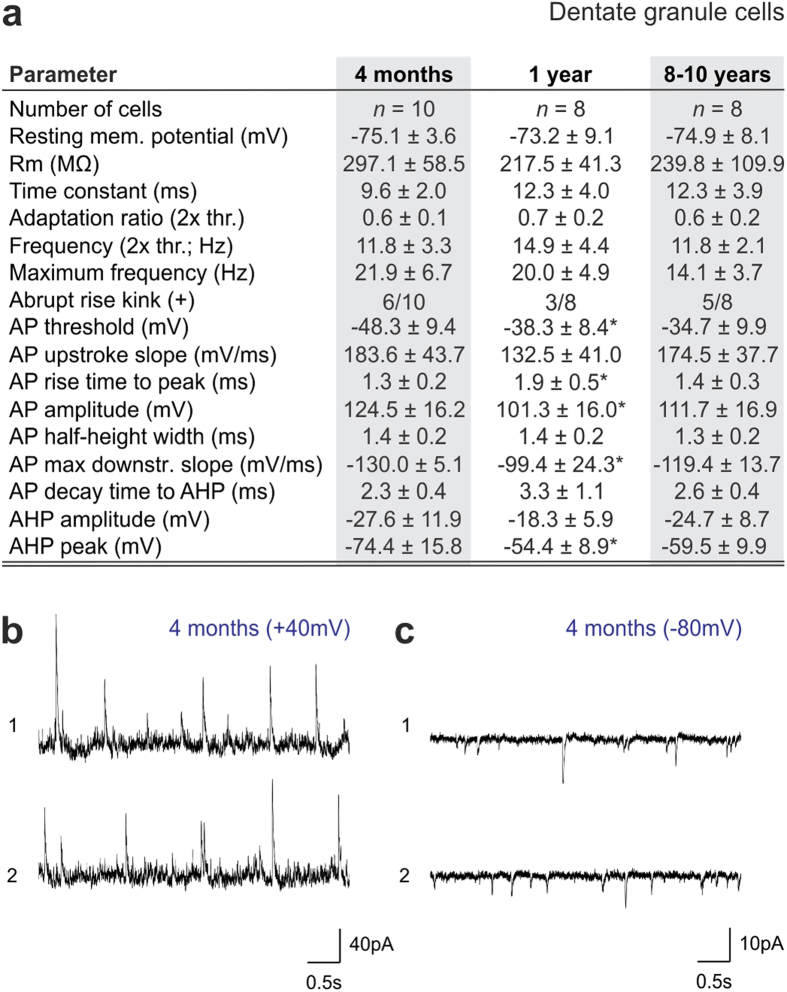
Biophysical parameters of dentate granule cells in naked mole rats. (**a**) Neurophysiological characteristics of dentate granule cells (*n *= 8–10 cells from a total of 3 animals/age). (**b,c**) Representative spontaneous activity traces obtained at +40 mV and −80 mV in 4-month-old naked mole rats reveals inhibitory and excitatory inputs, respectively. *p < 0.05 as compared to the 4-month-old naked mole rat.

Our characterization of GC excitability and intrinsic membrane properties by means of whole-cell patch-clamp electrophysiology (Supplementary Fig. 5a-b2) at successive time points (*n *= 10 [4 months], *n *= 8 [1 year], *n *= 8 [8–10 years]; Fig. 6e-g, Fig. 7a) support that cellular positioning is a major confound in population analysis since the frequency of APs, the contribution of the axon initial segment and somatic compartment to AP generation^46^ (space diagrams in Fig. 6e-g), and the reproducibility of AP amplitudes were location dependent. When comparing successive postnatal ages, we find that the maximum AP frequency, amplitude and their thresholds decrease over time (*p *< 0.05; 1 year vs. 4 months of age). Similarly, the slope of AP repolarization and the amplitude of AHP followed the same trend (Fig. 7a). GCs appeared most excitable at 1 year of age, with a trend towards reinstating early characteristics in aged animals. Finally, we show that GCs similar to CA1 pyramidal cells receive abundant and mixed GABA (Fig. 7b) and glutamate (Fig. 7c) synaptic inputs already at 4 months of age.

### Incomplete layer stratification and somatodendritic segregation of synapses

**Figure 8:**
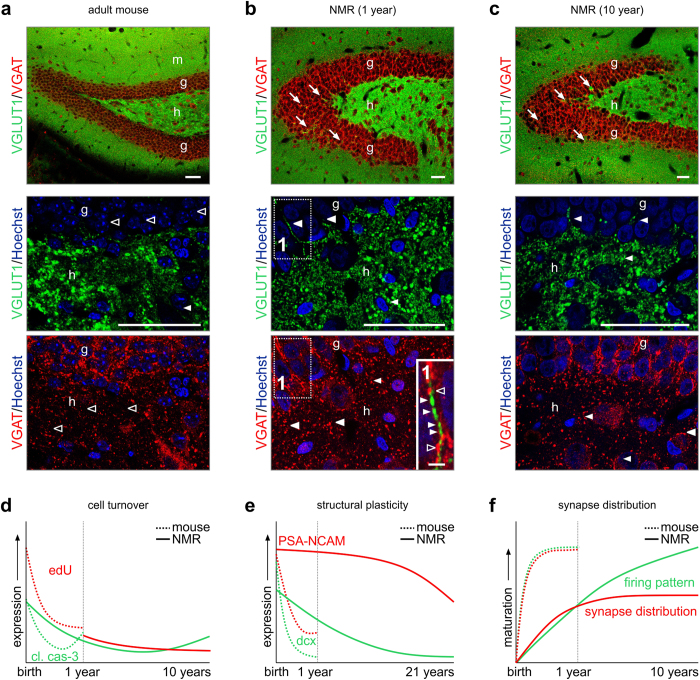
Synaptic maturation in the hippocampus of naked mole rats. (**a**) Inhibitory (VGAT^+^) and excitatory (VGLUT1^+^) terminals in the mouse hippocampus. Note the segregated distribution of both markers throughout the hippocampus. (**b–c**) In the naked mole rat, inhibitory and excitatory terminals are spatially less segregated, reminiscent of the immature mouse hippocampus. Note the presence of VGLUT1 in the granule cell layer (*arrows*), as well as the frequent intrusion of VGAT^+^ boutons in hilus (*arrowheads*) of the naked mole rat (*see also inset 1*). (**d–f**) Predicted developmental trajectories of cell turnover (**d**), structural plasticity (**e**) and synapse distribution (**f**) in aging mice and naked mole rats. *Abbreviations:* g, granule cell layer; h, hilus; m, molecular layer;; n, nucleus. *Scale bars *= 50 μm (**a,b,c**), 2 μm (inset 1).

The existence of mixed inhibitory/excitatory inputs onto both CA1 pyramidal cells and GCs prompted us to map whether the layer segregation of neurochemically distinct afferents follows morphological maturation. Therefore, we performed immunohistochemistry for vesicular glutamate transporter 1 (VGLUT1)^51^ and vesicular GABA transporter (VGAT)^52^. In the adult mouse dentate gyrus, we find the almost exclusive segregation of VGLUT1 and VGAT, with VGAT predominantly targeted towards the perisomatic domain of GCs^53^,^54^ (Fig. 8a). Likewise, VGLUT1 immunoreactivity predominated in the hilus (Fig. 8a, *lower panel*).

Even though the gross anatomical distribution of VGLUT1 and VGAT in 1- and 10-year-old naked mole rats resembled that of the mouse (Fig. 8b,c), high-resolution imaging showed that VGLUT1^+^ profiles frequently intruded into the GC layer (Fig. 8b, *lower panel*). Similarly, VGAT^+^ synapses failed to segregate in naked mole rats (Fig. 8b,c), with many found dispersed in the hilus of 1-, and to a lesser extent, 10-year-old naked mole rats. Overall, these data suggest that the segregation of excitatory and inhibitory terminals in the hippocampus occurs along year long time-scales in this species.

## Discussion

In this study, we show that brain maturation, as indicated by molecular, morphological, and electrophysiological features, is extremely protracted in naked mole-rats. Embryonic and early postnatal (pre-weaning) neurogenesis apparently provides adequate neuronal populations for life-long brain function in naked mole-rats, since cell proliferation rates as measured by EdU incorporation at postnatal dates, are not higher than in mice. This is also supported by the finding that markers of apoptosis are not elevated in the postnatal naked mole rat brain (Fig. 8d). Instead, we observed a prolonged retention of “immature” neuronal features including expression of PSA-NCAM, providing scaffolding for neurite outgrowth^33^,^34^,^55^ (Fig. 8e), delayed morphogenic maturation of hippocampal neurons, and incomplete synapse patterning as naked mole rats age (Fig. 8f). Therefore, we propose that while developmental neurogenesis provides adequate neuronal populations for adult brain functions, postnatal maturation of those neurons is greatly extended to provide much needed cellular dynamics to prevent structural damage and cell senescence in a low oxygen environment^2^.

We found lower amounts of neurogenesis in the adult naked mole rat, as compared to the mouse. This suggests that the bulk of neurons is produced during fetal development, and remains physiologically active until senescence. In common laboratory rodents, particularly mice, neuronal apoptosis peaks during the neonatal period to prune redundancy during brain circuit formation^56^. In our sample cohort, we did not find significant cleaved caspase-3 immunoreactivity during the period ranging from 7 days to 10 years postnatally, fuelling the provocative idea that cell production is tightly tuned by metabolic and/or O_2_ restrictions, likely limiting otherwise metabolically demanding processes of cell elimination. Alternatively, some neuronal cohorts might not reach full maturity even during the extended life-span of naked mole rats, thus precluding their incentive to initiate apoptotic programs. Instead, we find elevated cleaved caspase-3 levels in the 21-year-old naked mole rat, reflecting the age-related increase of apoptosis found in common laboratory rodents.

The regional organization of the naked mole rat brain is grossly similar to that of other rodents. Nevertheless, a key difference is in the olfactory system, where the layer stratification and cellular make-up of the piriform cortex resembles those of lower encephalisation index mammals. Analogous to the hedgehog brain, we demonstrate doublecortin immunoreactivity in layer II cells of the paleocortex, with their processes traversing layer III and targeted towards the underlying white matter. Based on these data, we concur with the hypothesis that doublecortin^+^ cells could represent a “dormant pool” of plastic cells in these animals^57^. Since doublecortin^+^ neuroblasts were found excitable in the rat piriform cortex earlier, we propose that retention of doublecortin/PSA-NCAM expression does not severely limit neuronal network functionality in naked mole rats.

Conspicuously, we find that the attainment of excitability in both CA1 pyramidal neurons and DGs seems uncoupled from their morphological complexity. This phenomenon is clearly different from corresponding cellular counterparts in mouse or rat, which show correlated molecular and morpho-functional differentiation^58^. A major change along the postnatal trajectory of both cell types studied here in the naked mole rat is the improved reproducibility of APs generated upon somatic current injections. This, even though indirectly, suggests that neuronal circuitries in naked mole rats gain functional precision over periods of years, rather than the conventional days-to-months seen in other laboratory species. In addition, APs were of higher peak amplitudes than in mouse (>40 mV difference). Although we did not address the molecular basis of this remarkable difference, we suggest that interspecies variations in the molecular structure, cellular composition and densities of Na^+^ channels might underscore this phenomenon. As such, Smith *et al*. (2011)^48^ found that Na_v_1.7 channels differ at key amino acid residues from those in mice and these Na_v_1.7 mutations were found associated with the lack of acid pain sensitivity in naked mole rats.

Although wild naked mole rats live under hypoxic conditions (~8% O_2_)^2^, our laboratory breeding colony was designed to keep naked mole rats under normoxic conditions (~20% O_2_). This set-up might have provoked modifications to neuronal excitability, e.g., disrupted Na^+^, K^+^ and Ca^2+^ channel function^59^,^60^,^61^,^62^,^63^. To fully answer the question if cellular turnover and neuronal excitability are regulated by metabolic and O_2_ restrictions, additional research should focus on diminished oxygen content in experimental setups to mimic hypoxic living conditions. Our analysis along extreme timescales and unusual metabolic complexity might have inherent limitations. For instance, the financial and time investment in keeping aging naked mole rats up to 21 years of age in laboratory settings, limits the availability of experimental tissues that can be obtained to reach sufficient animal numbers as compared to mouse studies. However, by using tissues from multiple ages (7 days, 3 and 5 months, 1, 4, 10 and 21 years) in complementary experimental designs, including proliferation assays, histochemistry and electrophysiology, we are confident to reach scientific validity to fully address our hypothesis from multiple angles in the developing naked mole rat.

In sum, we show that naked mole rats, a “treasure trove” for translational neurobiology, exhibit a very prolonged period of postnatal brain development consistent with a neotenous evolutionary mechanism. Protracted brain development may allow naked mole rat brain to cope with extremely low levels of O_2_ in their crowded subterranean burrows. Extended development may be accompanied by enhanced brain plasticity to preclude neurodegenerative processes during their extraordinary life-span. Thus, understanding the molecular basis of these processes warrants future research particularly aimed at expanding our tool kit to fight neurodegeneration and age-associated dementia.

## Methods

### Animals and tissue preparation for histochemistry

Embryonic (gestational day 18.5, *n *= 2), neonatal (postnatal day, *n *= 2) and adult C57Bl/6 N (*n *= 2; Charles River) mice were housed under standard laboratory conditions (12/12 h light cycle, 25% humidity). Fetal and neonatal mice were decapitated, their brains were removed from the skull and immersion fixed in 4% paraformaldehyde (PFA) in 0.1 M Na-phosphate buffer (PB, pH7.4) overnight. Adult male mice (2 - 3 months of age) were transcardially perfused under deep isoflurane anaesthesia by PFA-PB (0.1 M, pH 7.4) for 20 minutes and post-fixed over night at 4 degrees. Fetal and adult brains were cryoprotected in 30% sucrose in physiological saline for 48 h. Embryonic and adult mouse brains were cryosectioned coronally (Leica CV1850) at a thickness of 14 μm (onto SuperFrost^+^ glass slides) and 50 μm (free-floating), respectively.

Naked mole rats were born in a laboratory breeding colony in Chicago, IL, USA. The naked mole rats were non-breeding males with ages: 4 and 7 days, 3 and 5 months, and 1, 4, 10 and 21 years. Naked mole rats were housed under semi-natural conditions in an artificial burrow system within a colony room under normoxia. Because naked mole rats are poikilotherms (their internal temperature fluctuates considerably) their vivarium was maintained at 27.8 °C and 45–65% relative humidity. Younger animals were cooled and decapitated, and their brains immersion fixed in PFA-PB. Older naked mole rats were transcardially perfused with 4% PFA-PB for 20 minutes and post-fixed over night at 4 degrees. Whole brains were cryoprotected as above. Neonatal and adult brains were cryosectioned coronal (Leica CV1850) at 14 μm thickness as glass-mounted specimens (SuperFrost^+^) and 50 μm thickness as free-floating sections collected in 0.1 M PB, containing 0.1% sodium azide, respectively, and validated to produce comparable data quality (Supplementary Fig. 2e,f).

Experimental procedures on mice were approved by the regional ethical committee (Stockholms Norra Djurförsöksetiska Nämnd; #N512/12). All experiments on naked mole rats were approved by the University of Illinois at Chicago Institutional Animal Care and Use Committee, in accordance with the National Institutes of Health guidelines. Particular effort was directed to minimize the number of animals and their suffering during the experiments.

### Click-iT EdU labeling

Adult male mice (6–8 weeks old; *n *= 4) and naked mole rats (aged 1, 4 and 10 years; *n *= 2, 3, 2, respectively) were injected intraperitoneal for 7 days with EdU dissolved in saline (20 mg/kg)^27^,^28^. We preferred a repeated EdU protocol since neither the efficacy of EdU absorption upon i.p. or s.c. injection nor its access to and clearance from the CNS of naked mole rats is known. As a positive control, we exposed embryonic day (E)14.5 mice to a single dose of EdU and assessed EdU incorporation on E18.5. As expected, this experiment showed abundant EdU incorporation throughout the fetal mouse hippocampus (Supplementary Fig. 2a), coinciding with the vast expansion of this structure during late-gestation.

Tissues were taken either one day or 8 weeks post EdU. Mice were deeply anaesthetized with isofluran, while naked mole rats were anaesthetized with intraperitoneal pentobarbital and subsequently decapitated. Brains were removed and immersion fixed in 4% PFA-PB, cryoprotected in 30% sucrose for 48 hours and sectioned sagittal (50 μm) on a cryostat microtome (Leica CV1850) to reveal the complete rostral migratory stream (Fig. 1a). EdU was visualized with Alexa 555-azide using Click-iT labeling (Life Technologies). Tissues were treated with Sudan black (5 minutes) to quench autofluorescence and reveal specific EdU labeling (Supplementary Fig. 2b) while Hoechst 33,342 was used to reveal tissue-architecture. An E18.5 mouse embryo, exposed to a single maternal injection of EdU (33 mg/kg) at E14.5 and immersion fixed at E18.5, was used as a positive control (Supplementary Fig. 2a).

### Western blotting

Brain tissues from mouse olfactory bulb and neocortex, naked mole rat neocortex and entire forebrain were homogenized in TNE buffer containing 0.5% Triton X-100 (Sigma), 1% octyl-β-D-glucopyranoside (Calbiochem), 5 mM NaF, 100 μM Na_3_VO_4_ and a cocktail of protease inhibitors (Complete^TM^, Roche) by ultrasonication and lysed for 45 minutes at 4 ^o^C. Cell debris and nuclei were removed by centrifugation (800 *g*, 10 min at 4 ^o^C). Protein concentrations were determined by Bradford’s colorimetric method. Samples were diluted to a final protein concentration of 2 μg/μl, denatured in 5x Laemmli buffer, and analyzed by SDS-PAGE on 8% or 10% resolving gels. After transferring onto Immobilon-FL polyvinylidene difluoride membranes (Millipore), protein samples on membranes were blocked in Odyssey blocking buffer (Li-Cor Biosciences; 1 h) and exposed to primary antibodies (Supplementary Fig. 1b) overnight at 4 ^o^C. Appropriate combinations of IRDye-800CW and IRDye-680-conjugated secondary antibodies were used for signal detection (Li-Cor Biosciences; from goat or rabbit hosts; 1:10,000, 2 h). Image acquisition and analysis were performed on a Li-Cor Odyssey IR imager. β-Actin (1:10,000; Sigma) served as loading control.

### Multiple immunofluorescence labeling

Serial glass-mounted or free-floating sections were processed according to published histochemical protocols, including the moderation of tissue autofluorescence (by using Autofluorescence Eliminator Reagent (Millipore) in specimens from 1, 4, 10 and 21 year-old naked mole rats) and signal enhancement. In brief, sections were rinsed in 0.1 M PB (PH 7.4) and preincubated with 5% normal donkey serum (NDS; Jackson Immunoresearch), 2% bovine serum albumin (BSA; Sigma) and 0.3% Triton X-100 in 0.1 PB for 1 h at 22-24 ^o^C. Sections were then exposed to select cocktails of primary antibodies (Supplementary Fig. 1b) in 0.1 M PB, to which 0.1% Triton X-100, 0.1% BSA and 1% NDS had been added, for 48 h at 4 °C. Hoechst 33,342 (Sigma), a nuclear dye, was routinely used to reveal tissue cytoarchitecture. All markers were visualized with combinations of carbocyanine (Cy)2, Cy3 or Cy5-conjugated antibodies (1:300–1:800 dilutions) generated in donkey (Jackson; 2% BSA in 0.1 M PB, 2 h at 22-24 ^o^C). After secondary antibody, tissues were washed once with 70% ethanol (5 min) and subsequently incubated with Autofluorescence Eliminator Reagent for 5 minutes at room temperature to eliminate false positive signals due to the age-associated deposition of lipofuscin^64^ (Supplementary Fig. 2b). Tissues were washed twice with 70% ethanol to remove excess Reagent and transferred to PB containing Hoechst 33,342 for 10 minutes. Free-floating sections were mounted from water onto fluorescence-free glasses. All sections were coverslipped by Aquamount fluorescence mounting medium (DAKO).

### Conventional and laser-assisted fluorescence microscopy

Scaled brain overviews (Supplementary Fig. 1a) were acquired using a Bio-Rad XRS^+^ imaging platform. Serial sections were first inspected on a Nikon FXA fluorescence microscope, and survey images captured at 4x primary magnification using a CoolSnap HQ^2^ CCD camera and QImage analysis software (Photometrics). Next, select slides were analyzed on a 710LSM confocal laser-scanning microscope (Carl Zeiss). Emission spectra for each dye were limited as follows: Hoechst (420–480 nm), carbocyanine 2 (Cy2)/DyLight488 (505–530 nm), Cy3/DyLight549 (560–610 nm), and Cy5(DyLight649 (650–700 nm). Co-localization of select histochemical marker pairs was defined as immunosignals being present without physical signal separation in ≤1.0 μm optical slices at 40× (Plan-Neofluar 40×/1.30) or 63× (Plan-Apochromat 63×/1.40) primary magnification, and verified by capturing serial orthogonal *z* image stacks at 63x primary magnification (pinhole: 30 μm, 2048 × 2048 pixel resolution). Image surveys were generated using the tile scan function with optical zoom ranging from 0.6x to 1.5x at 10x primary magnification (objective, EC Plan-Neofluar 10×/0.30). Fluorescence intensity distribution for PSA-NCAM (“heat” maps; Supplementary Fig. 3) was presented as five categories with increasing color intensity corresponding to a higher extent of area coverage for the fluorescence signal. Doublecortin-positive (DCX^+^) neurons and their leading/trailing processes were depicted in green color throughout the schematic brain illustrations (Supplementary Fig. 3). Antibody penetration was verified by obtaining a *z*-stack vertically spanning the tissue depth at 63x (1.5 zoom) of GFAP-immunolabelling in the fimbria hippocampi of glass-mounted (14 μm; Supplementary Fig. 2e) and free-floating sections (50 μm; Supplementary Fig. 2f). No differences in staining intensity due to tissue preparation were observed. Images were processed using the ZEN2011 software (Carl Zeiss). Multipanel figures were assembled in CorelDraw X5 (Corel Corp).

### Patch-clamp electrophysiology

Naked mole rats of 4 months, 1 year and 8–10 years old were used for *in vitro* electrophysiology (*n *= 3 animals per age). Transverse slices of 300 μm were cut with a vibrotome (Leica VT1200S) from the dorsal hippocampus in ice cold slicing artificial cerebrospinal fluid (aCSF) which contained in mM: 90 NaCl, 2.5 KCl, 1.25 Na_2_HPO_4_, 0.5 CaCl_2_, 8 MgSO_4_, 26 NaHCO_3_, 20 D-Glucose, 10 HEPES, 3 Na-pyruvate, 5 Na-ascorbate. Slices were then moved for 10–15 min into room temperature (RT) recovery solution which contained in mM: 93 NMDG, 93 HCl, 2.5 KCl, 1.25 Na_2_HPO_4_, 0.5 CaCl_2_, 8 MgSO_4_, 26 NaHCO_3_, 20 D-Glucose, 10 HEPES, 3 Na-pyruvate, 5 Na-ascorbate. Slices were incubated at RT for 60 min in recording aCSF which contained in mM: 124 NaCl, 2.5 KCl, 1.25 Na_2_HPO_4_, 2 CaCl_2_, 2 MgSO_4_, 26 NaHCO_3_, 10 D-Glucose (all from Sigma), all the solutions were pH set to 7.4 and bubbled with carbogen gas (5% CO_2_ / 95% O_2_). The perfusion rate of slices in the recording chamber was 4–6 ml/min. Recordings were carried out at RT with borosilicate glass electrodes (WPI) of 3–5 MΩ (P-97, Sutter). Electrodes were filled with intracellular solution (pH 7.4), which contained in mM: 135 K-gluconate, 10 KCl, 10 HEPES, 4 ATP-Mg, 0.3 GTP-Na, 5 EGTA and 0.5 mg/ml biocytin (Sigma). Whole-cell patch clamp recordings were made using Axopatch 200B (Molecular Devices) and data were digitized at 20 KHz (Digidata 1440 A and pClamp 10.4; Molecular Devices). In the case of >20% change of access resistance, data from the given cell were discarded. Action potential (AP) threshold was defined as the voltage where the slope trajectory reached 10 mV/ms. AP amplitude was defined as the difference in membrane potential between threshold and peak. Afterhyperpolarization (AHP) amplitude was defined as the difference between threshold and the most negative membrane potential attained during the AHP. These parameters were measured for the first AP elicited by depolarizing 750-ms-long current step of amplitude just sufficient to bring the cell to threshold for AP generation. Adaptation ratio was calculated as the ratio of the average of the last two interspike intervals relative to the first interspike interval during a 750-ms-long current pulse of double the amplitude, which elicited a just suprathreshold response. Firing frequency was calculated from the number of spikes of the same spike train. All electrophysiological parameters were measured in pClamp or applying procedures written in Matlab. Voltage clamp recordings to reveal the presence of excitatory and inhibitory synaptic currents were recorded at holding potentials +40 mV and −80 mV in 4-month-old naked mole rats. Sample traces show ≤5 s epochs of these recordings.

### *Post-hoc* neuroanatomy for cell reconstruction

Hippocampal brain slices used for electrophysiology were post-fixed in 4% paraformaldehyde in phosphate-buffer (PB, 0.1 M, pH 7.4) overnight at 4 ^°^C. Slices were repeatedly washed in PB and blocked for 3–4 h at room temperature (RT) with a mixture of 2.5% bovine serum albumin (BSA), 2.5% normal goat serum (NGS) and 0.5% Triton X-100 in PB. Biocytin filled neurons were visualized with carbocyanine (Cy)3-tagged streptavidin (1:500; Jackson-Immunoresearch, West Grove, PA) diluted in 2% BSA and 1% Triton X-100 in PB and incubated overnight at 4 °C. After Sudan-black quenching of autofluorescence and extensive washing in PB, slices were mounted on glass slides in glycerol-gelatin (Sigma). Analysis of specimens was performed using confocal laser-scanning microscopy (LSM780, Zeiss) and Zen 10.0 software. Three-dimensional filaments of biocytin filled cells from Z-stack images were reconstructed in Imaris 7.5.4. Statistical analysis of dendritic data was also performed with Imaris. Care was taken to define process endings and avoid measuring artifacts through cut surfaces due to slice preparations.

### Comparative topography and nomenclature

To compare the distribution of immunoreactive structures at the level of neuronal perikarya, processes and synapses, mouse and naked mole rat tissues were scanned at identical pinhole (30–75 μm at 20x or 40x primary magnification), detector gain and offset settings, and processed simultaneously. Distribution maps were drawn on regionalized schemas from the brain atlases of Paxinos and Franklin^65^ and Xiao and colleagues^66^ for mouse and naked mole rat, respectively. In developmental studies, we relied on the nomenclature introduced by Ashwell and Paxinos^67^ for fetal and neonatal mouse brain. For the adult naked mole rat, we adopted the nomenclature by Xiao *et al*.^66^ unless stated otherwise.

### Statistical analysis

Statistical significance for all parameters (Figs 5,7) was tested with a nonparametric Mann-Whitney U-test. Significance was accepted at *p *< 0.05.

## Additional Information

**How to cite this article**: Penz, O. K. *et al*. Protracted brain development in a rodent model of extreme longevity. *Sci. Rep*. **5**, 11592; doi: 10.1038/srep11592 (2015).

## Supplementary Material

Supplementary Information
