# Supplementary material for: Protracted brain development in a rodent model of extreme longevity
Source: Sci Rep. 2015 Jun 29;5:11592. doi: 10.1038/srep11592 (PMC4484490; doi:10.1038/srep11592)
Supplement: Supplementary Information [file srep11592-s1.pdf]

Protracted brain development in a rodent model of extreme longevity

Running title: Delayed brain development of the naked mole rat

Orsolya K. Penz[1,*], Janos Fuzik[1,2,*], Aleksandra B. Kurek[2], Roman Romanov[1], John Larson[3,4,5], Thomas J. Park[3,4], Tibor Harkany[1,2,&,#] & Erik Keimpema[1,2,&,#]

[1]Division of Molecular Neurobiology, Department of Medical Biochemistry & Biophysics, Scheeles väg 1:A1, Karolinska Institutet, SE-17177 Stockholm, Sweden; [2]Department of Molecular Neurosciences, Center for Brain Research, Medical University of Vienna, Spitalgasse 4, A-1090 Vienna, Austria; [3]Department of Biological Sciences, [4]Laboratory for Integrative Neuroscience, University of Illinois, 840 West Taylor Street, Chicago, IL 60607, USA, and [5]Department of Psychiatry, University of Illinois College of Medicine, 1601 West Taylor Street, Chicago, IL 60612.

*O.K.P. and J.F. contributed equally to this study.

[&]E.K. and T.H. share senior authorship

[#]*Address correspondence* to: Tibor Harkany (at the Karolinska Institutet); *e-mail*: Tibor.Harkany@ki.se or Erik Keimpema (at the Medical University of Vienna); *e-mail*: Erik.Keimpema@meduniwien.ac.at

This file contains:

- *Supplementary Figures 1 - 5*
- *Legends to Supplementary Figures*
- *Supplementary References*

Legends to Supplementary Figures

Figure S1 Brain morphology and antigen confirmation in naked mole rats. (**a**) Brain morphology and size of neonatal and adult C57Bl6/N mice. (**a$_1$**) Protracted brain growth in naked mole rats aged from 4 days to 21 years. All brains were scaled to those in (a), and photographed on a millimeter-scaled background. Note that postnatal brain development continues to at least 1 year of age in naked mole rats. (**b**) List of markers used for immunofluorescence labeling and Western blotting. Antibodies used in this study to investigate the localization and identity of neuronal and glial populations in the brains of mice and naked mole rats were listed, including working dilutions and suppliers. Anti-VGLUT1 antibody from guinea-pig hosts was made available by H. Hioki and T. Kaneko. The specificity of these antibodies were previously extensively characterized, including the use of knock-out mice and pre-adsorption with respective immunizing antigen[1]. (**c**) Representative Western blots showing equivalent molecular weights of target proteins from mouse and naked mole rats probed with antibodies listed in (b). β-actin was used to probe for equivalent protein loading in the various samples used for Western blotting. *Abbreviations*: cr, cerebellum; ctx, cerebral cortex; forebr., forebrain; NMR, naked mole rat; ob, olfactory bulb.

Figure S2 Cell proliferation in the naked mole rat. (**a**) EdU$^+$ cells labeled in the E18.5 mouse embryonic hippocampus, pulsed with a single EdU injection at E14.5. (**b**) Auto-fluorescent eliminator (A.F. eliminator) quenched auto-fluorescence (*open arrowheads)* found in unlabeled tissues but did not affect fluorescence after Click-iT labeling (*closed arrowheads*). Note that auto-fluorescent cells bleed through in both channels. (**c**) Ki67$^+$ cells were found in naked mole rat progenitor proliferation areas, including the svz-rms-ob axis. (**d,e**) Complete *z*-stacks spanning the entire depth of the tissues of GFAP immunofluorescence in the fimbria hippocampi of 5 months old (glass mounted, 14 µm; d) and 21 year old (free-floating, 50 µm; e) naked mole rat sections show similar antibody penetration regardless of tissue processing and thickness. Note that auto-fluorescence quenching shrinks tissue thickness due to dehydration. *Abbreviations:* CA1, cornu ammonis 1; CPu, caudate putamen; ctx, cortex; h, hilus; lv, lateral ventricle; ob, olfactory bulb; rms, rostral migratory stream; sgz, subgranular zone; svz, subventricular zone. *Scale bars* = 50 µm (a,b,c), 10 µm (d), 5 µm (e,f).

Figure S3 Comparative topological mapping of DCX$^+$ cells and processes immunoreactive for PSA-NCAM in juvenile and adult mice and naked mole rats. (**a-b$_1$**) Serial sections from both species were processed simultaneously. The nomenclature introduced by Paxinos *et al.* for mice[2] and by *Xiao et al.* for naked mole rats[3] was used. Coordinates indicate distances from bregma. Green ovals and processes denote the localization of somata and leading/trailing processes of doublecortin (DCX)-positive cells, respectively. PSA-NCAM immunoreactivity was scaled from deep orange (high expression) to white (negative) by an examiner blinded to the age of the particular animals. Labeling patterns in mice at both ages correspond to data published[4]. *Abbreviations*: 3v, third ventricle; ac, anterior commissure; AO, anterior olfactory nucleus; AOD, anterior olfactory nucleus, dorsal; AOV, anterior olfactory bulb, ventral; APT, anterior pretectal nucleus; aq, acqueduct; arc, arcuate nucleus; av, anteroventral thalamic nucleus; bla, basolateral amygdaloid nucleus, anterior; bhp, basolateral amygdaloid nucleus posterior; CA1-3, Cornu Ammonis subfields 1-3; cc, corpus callosum; CG, cingulum; cing, cingulate cortex; cp, cerebral peduncule; CPu, caudate putamen; Ctx, cortex; DG, dentate gyrus; EPL, external plexiform layer; GL, glomerular layer; gp, globus pallidus; GRL, granular layer; hc, hippocampus; HDB, horizontal diagonal band of Broca; ic, internal capsule; itr, internal thalamic radiation; LEnt, lateral entorhinal cortex; LO, lateral orbital cortex; LGC, lateral geniculate complex; lv, lateral ventricle; MG, medial geniculate nucleus; MM, medial mammillary nucleus; MS, medial septum; ov, olfactory ventricle; PG, periaqueductal grey; pir, piriform cortex; PLCo;, posterolateral cortical amygdaloid nucleus; PM, premammillary nucleus; PV, paraventricular thalamic nucleus; rms, rostral migratory stream; RN, red nucleus; s, subiculum; sctx, somatosensory cortex; SN(r), substantia nigra (pars reticulate); SuM, Supramammillary nucleus; VGC, ventral geniculate complex; vmh, ventromedial hypothalamus; VO, ventral orbital cortex.

Figure S4 Structural plasticity in the rostral migratory stream of naked mole rats. (**a-c**) DCX$^+$/PSA-NCAM$^+$ migrating neurons (*arrows*) populate the rostral migratory stream in the neonatal and adult mouse brain. Note that DCX$^+$ fibers radiate past secretagogin$^+$ neurons[5,6], into the granule layer towards the glomeruli (c, inset 1). (**d**) Similar, neonates and 5-month-old naked mole rats showed DCX$^+$/PSA-NCAM$^+$ migrating neurons (*arrows*) in the rostral migratory stream towards the olfactory bulb. (**e,f**) In contrast to mice (b,c), DCX was highly expressed in the olfactory bulb of the 1 year old naked mole rat. Note the reduced amount of secretagogin$^+$ cells in the granule layer of naked mole rats (f). (**g,h**) In aged naked mole rats, neurogenesis was reduced with sporadic

DCX$^+$ neurons present in the 21-year-old olfactory bulb. Comparable to the hippocampus, PSA-NCAM immunoreactivity remained in the adult olfactory bulb (inset 5). Numbered boxes denote insets. *Scale bars* = 100 µm (f,g), 40 µm (a,b,c,d,e,h), 10 µm (1,2,3,4,5).

Figure S5 Biophysical properties and dendrite morphology of a dentate granule cell in the 4-month-old naked mole rat. (**a**) Representative current-clamp recording of a CA1 pyramidal cell in the mouse hippocampus. Note that the peak AP current corresponds to the calculated Na^+ reversal potential. (**b,b$_1$**) Current clamp recordings of a reconstructed granule cell (b_1) with the most hyperpolarizing and depolarizing current steps depicted in black. Membrane potential traces shown in red were evoked by a depolarizing current step at 2x threshold of the cells (in red). (**b$_2$**) Phase diagram of 2x action potential threshold stimulus. The first action potential phase diagram was colored red with subsequent traces progressively shaded from warm to cool colors, ending in blue.

References

1. Kaneko, T., Fujiyama, F., & Hioki, H. Immunohistochemical localization of candidates for vesicular glutamate transporters in the rat brain. *J Comp Neurol* **444**, 39-62 (2002).
2. Paxinos,G. & Franklin,K.B.J in The Mouse Brain in Stereotactic Coordinates (Gulf Professional Publishing, 2004).
3. Xiao, J., Levitt, J.B., & Buffenstein, R. A stereotaxic atlas of the brain of the naked mole-rat (Heterocephalus glaber). *Neuroscience* **141**, 1415-1435 (2006).
4. Nacher, J., Crespo, C., & McEwen, B.S. Doublecortin expression in the adult rat telencephalon. *Eur. J Neurosci.* **14**, 629-644 (2001).
5. Mulder, J. *et al.* Secretagogin is a Ca2+-binding protein specifying subpopulations of telencephalic neurons. *Proc. Natl. Acad. Sci. U. S. A* **106**, 22492-22497 (2009).
6. Attems, J. *et al.* Clusters of secretagogin-expressing neurons in the aged human olfactory tract lack terminal differentiation. *Proc. Natl. Acad. Sci. U. S. A* **109**, 6259-6264 (2012).

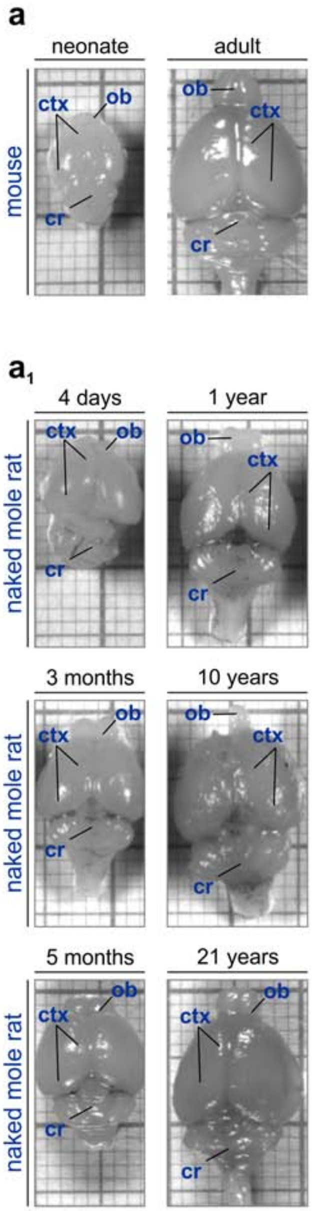

b

antibody	host	histochemistry	WB	catalogue #	supplier
Cleaved caspase-3	rabbit	1:500		9664	Cell Signaling Technology
Doublecortin	goat	1:200	1:500	SC-8066	Santa Cruz Biotechnology
GFAP	mouse	1:2,000	1:1,000	MAB3402	Millipore
Ki67	rabbit	1:500		AB9260	Millipore
L1-NCAM	rat	1:2,000		MAB5272	Millipore
Solanum t. lectin		1:100		B-1165	Vector
Nestin	mouse	1:500		MAB353	Millipore
PSA-NCAM	mouse	1:400		MAB5324	Millipore
Secretagogin	rabbit	1:200	1:500		Dr. L. Wagner
VGAT	rabbit	1:500	1:1,000	131003	Synaptic Systems
VGLUT1	guinea pig	1:500	1:1,000		Dr. T. Kaneko

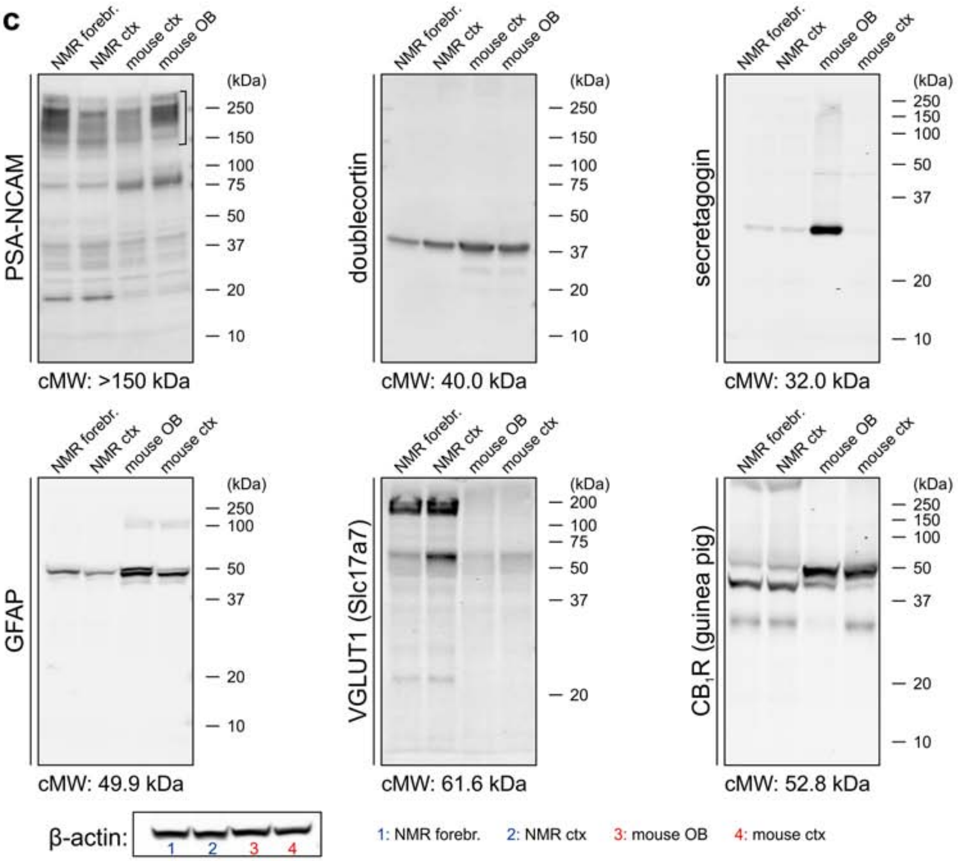

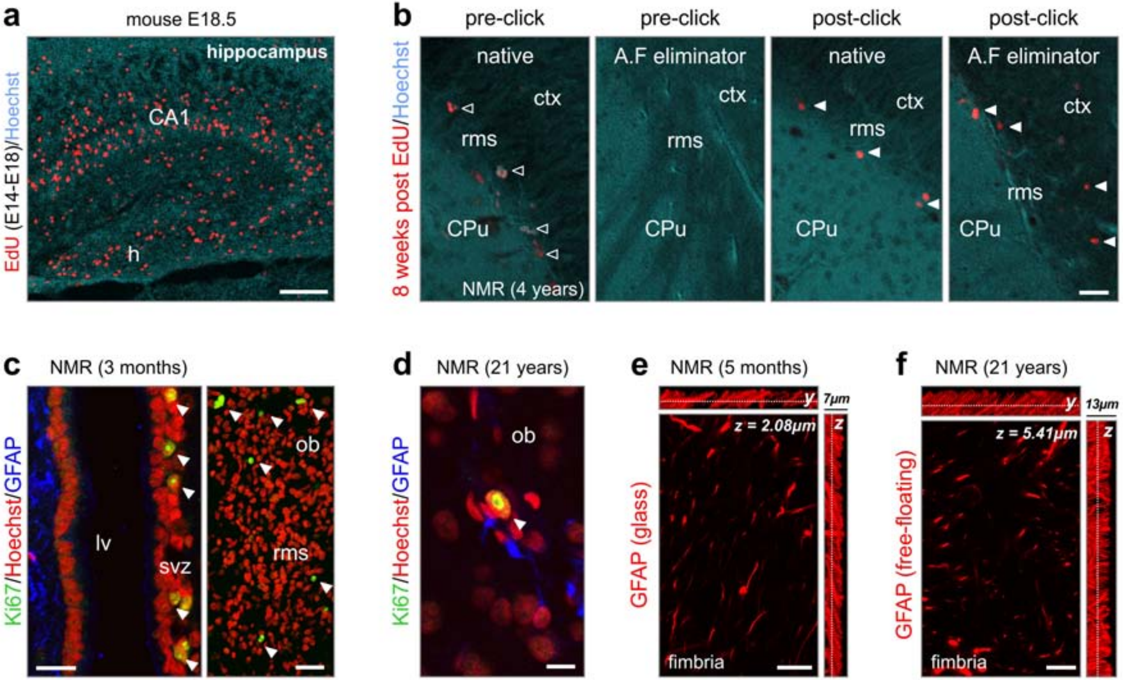

a
mouse E18.5
hippocampus
CA1
h
EdU (E14-E18)/Hoechst
b
pre-click
pre-click
post-click
post-click
native
A.F eliminator
native
A.F eliminator
ctx
rms
CPu
NMR (4 years)
8 weeks post EdU/Hoechst
c
NMR (3 months)
lv
svz
ob
rms
Ki67/Hoechst/GFAP
d
NMR (21 years)
ob
Ki67/Hoechst/GFAP
e
NMR (5 months)
y
7μm
z = 2.08μm
z
GFAP (glass)
fimbria
f
NMR (21 years)
y
13μm
z = 5.41μm
z
GFAP (free-floating)
fimbria

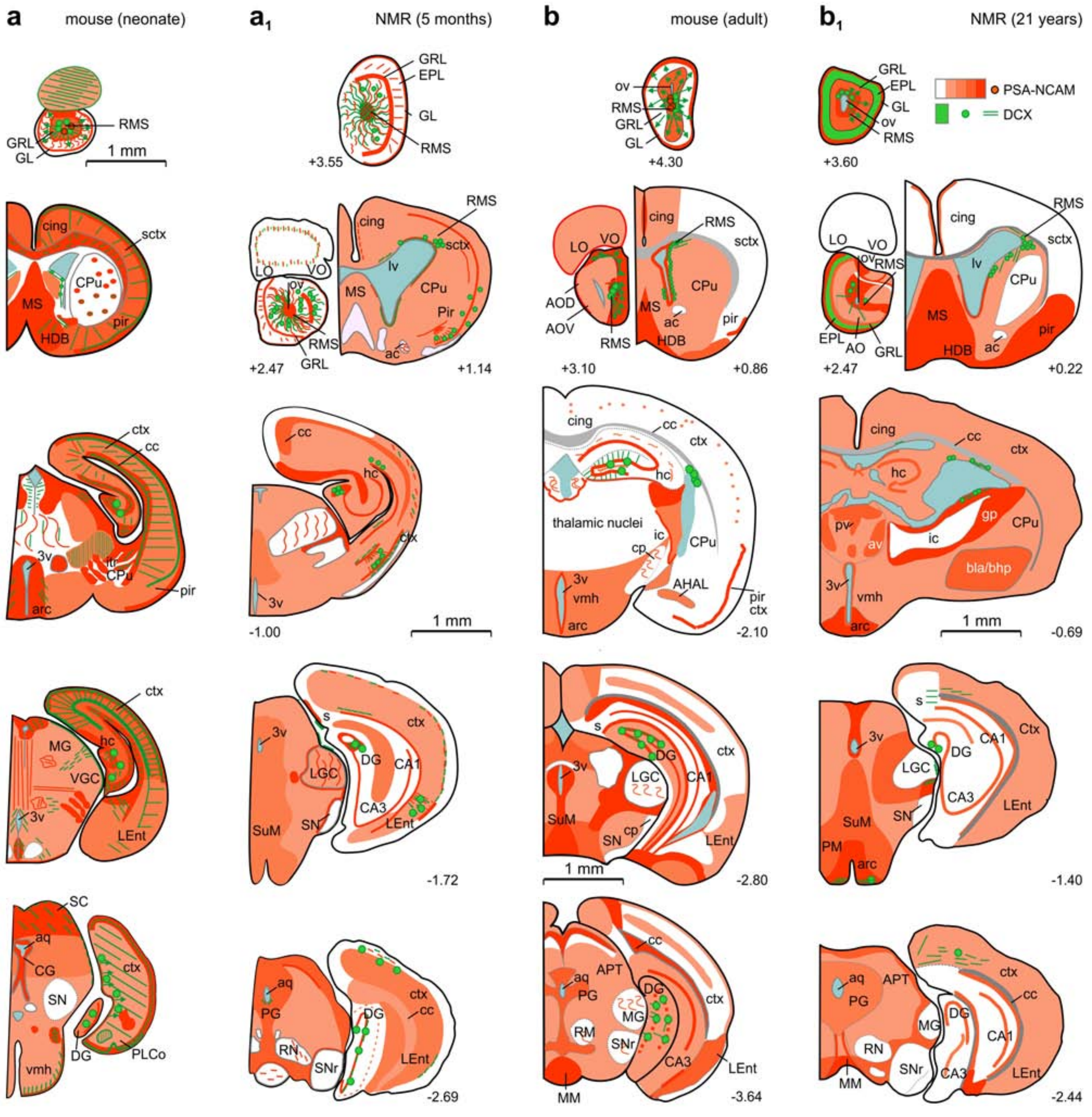
a
mouse (neonate)
a1
NMR (5 months)
b
mouse (adult)
b1
NMR (21 years)
PSA-NCAM
DCX
GRL
EPL
GL
RMS
ov
1 mm
+3.55
+4.30
+3.60
+2.47
+1.14
+3.10
+0.86
+0.22
-1.00
-2.10
-0.69
-1.72
-2.80
-1.40
-2.69
-3.64
-2.44
cing
sctx
CPu
MS
HDB
pir
LO
VO
lv
Pir
ac
AOD
AOV
AO
ctx
cc
hc
3v
itr
arc
thalamic nuclei
ic
cp
vmh
AHAL
pir ctx
pv
av
gp
bla/bhp
MG
VGC
LEnt
s
DG
CA1
CA3
LGC
SN
SuM
PM
Ctx
SC
aq
CG
PLCo
PG
RN
SNr
APT
RM
MM

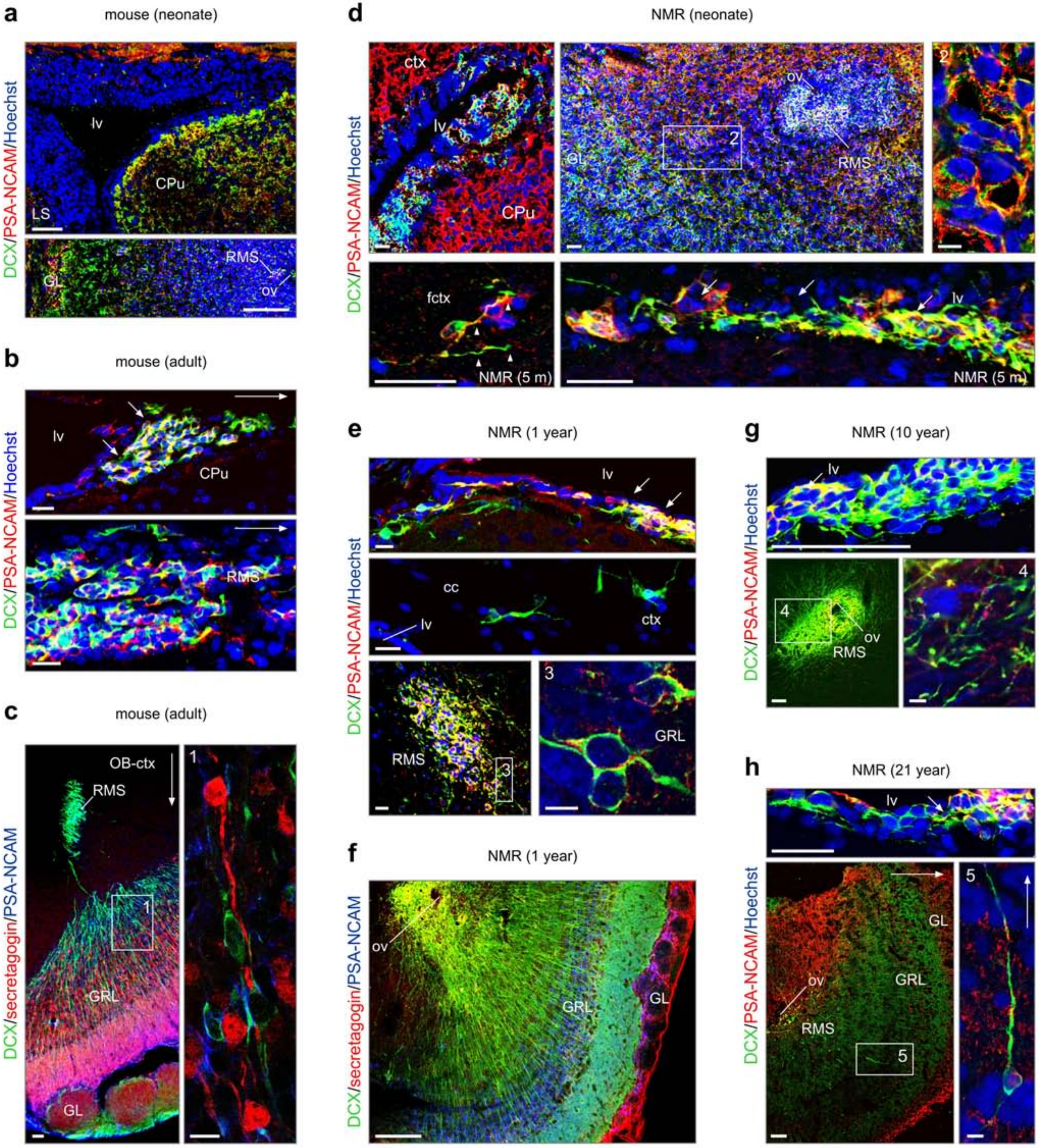
a
mouse (neonate)
DCX/PSA-NCAM/Hoechst
lv
CPu
LS
GL
RMS
ov
b
mouse (adult)
DCX/PSA-NCAM/Hoechst
lv
CPu
RMS
c
mouse (adult)
DCX/secretagogin/PSA-NCAM
OB-ctx
RMS
1
GRL
GL
d
NMR (neonate)
DCX/PSA-NCAM/Hoechst
ctx
lv
CPu
ov
2
GL
RMS
fctx
NMR (5 m)
lv
NMR (5 m)
e
NMR (1 year)
DCX/PSA-NCAM/Hoechst
lv
cc
lv
ctx
RMS
3
GRL
f
NMR (1 year)
DCX/secretagogin/PSA-NCAM
ov
GRL
GL
g
NMR (10 year)
DCX/PSA-NCAM/Hoechst
lv
4
ov
RMS
h
NMR (21 year)
DCX/PSA-NCAM/Hoechst
lv
5
GL
GRL
ov
RMS
5

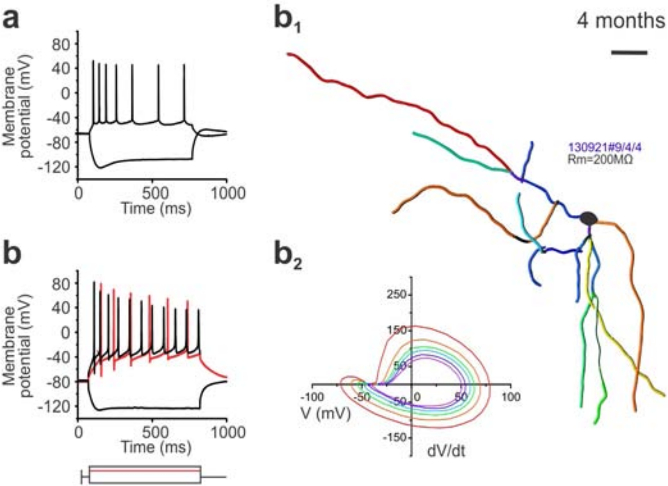
a
80
40
0
-40
-80
-120
Membrane potential (mV)
0
500
1000
Time (ms)
b
80
40
0
-40
-80
-120
Membrane potential (mV)
0
500
1000
Time (ms)
b1
4 months
130921#9/4/4
Rm=200MΩ
b2
250
150
50
-50
-150
-100
-50
0
50
100
V (mV)
dV/dt
